# Occupational Sun Exposure and Skin Neoplasms in a Dermatology Referral Service in Southeast Brazil: A Cross-Sectional Study

**DOI:** 10.7759/cureus.107750

**Published:** 2026-04-26

**Authors:** Maria Eduarda Augusto Cardozo, Flávia Regina Ferreira, Mariana Ferreira da Silva, Polyana Guedes dos Santos, Julia de Jesus Pereira de Andrade

**Affiliations:** 1 Department of Medicine, University of Taubaté, Taubaté, BRA; 2 Department of Dermatology, Hospital Municipal Universitário de Taubaté, Taubaté, BRA; 3 Department of Medicine, Humanitas - Faculty of Medical Sciences, São José dos Campos, BRA

**Keywords:** health behavior, occupational exposure, skin neoplasms, sunlight, sunscreening agents, ultraviolet rays

## Abstract

Background: Skin neoplasms, including melanoma and non-melanoma types, represent a major public health concern in Brazil. Occupational sun exposure has been identified as an important risk factor, particularly among outdoor workers. This study aimed to describe the characteristics of individuals with skin neoplasms in a hospital-based sample from a dermatology referral service, and to characterize their epidemiological profile and patterns of sun exposure.

Methods: This exploratory, cross-sectional, descriptive, hospital-based study was conducted at a dermatology referral outpatient clinic between December 2024 and June 2025. A non-probabilistic convenience sample of adult patients (≥18 years) attending the clinic during the study period was included. For analytical purposes, only individuals with a confirmed current or previous diagnosis of skin neoplasms were considered. Data were collected using a structured questionnaire developed by the authors to assess demographic characteristics, occupational sun exposure, photoprotection practices, and personal and family history of skin neoplasms. Descriptive statistical analysis was performed.

Results: A total of 120 individuals were assessed during the study period. Of these, 33 (27.5%) had a confirmed diagnosis of skin neoplasms, while the remaining 87 (72.5%) presented with other dermatological conditions. Among individuals with skin neoplasms, 18 (54.5%) were male, and 30 (91.0%) self-identified as White. Most participants were older than 55 years at the time of assessment (27, 82.0%), and 17 (51.6%) received their first diagnosis after 55 years of age. Low educational level was observed, with 14 (42.4%) not having completed primary education. Daily occupational sun exposure was reported by 22 (66.6%) participants, most frequently between 10 a.m. and 4 p.m. (n=16; 48.5%). Daily sunscreen use was reported by 12 (36.3%), whereas 14 (42.0%) reported no use. Non-melanoma skin neoplasms, particularly basal cell carcinoma, were the most frequent histological types, whereas melanoma was less frequently observed.

Conclusions: This study provides a characterization of individuals with skin neoplasms in a hospital-based sample from a dermatology referral service, including their epidemiological profile and patterns of sun exposure. In this sample, skin neoplasms were observed predominantly among older individuals with fair skin, lower educational levels, and a history of outdoor work. Low adherence to photoprotection practices was also identified. These findings highlight patterns in this setting and may support future research and preventive strategies.

## Introduction

Carcinogenesis is a complex process involving the accumulation of genetic alterations in combination with environmental factors, such as ultraviolet (UV) radiation exposure. This process involves the malignant transformation of cells driven by mutations and epigenetic changes [1]. Skin neoplasms, including melanoma and non-melanoma types, represent a major public health concern worldwide. According to the American Academy of Dermatology (AAD, 2020), non-melanoma skin neoplasms, basal cell carcinoma (BCC) and squamous cell carcinoma (SCC), as well as melanoma, are more frequently observed in body areas with higher UV exposure [2].

Public underestimation of the harmful effects of UV radiation contributes to the high prevalence of skin neoplasms, as excessive sun exposure is a leading cause of photodamage to cutaneous cells. Skin neoplasms, especially non-melanoma types, are among the most common malignancies and have high clinical relevance [3]. Progressive depletion of the ozone layer, which filters UV radiation, further increases the incidence of skin neoplasms; a 10.0% reduction in ozone levels has been estimated to result in approximately 300,000 new non-melanoma cases and 4,500 melanoma cases annually [4]. In Brazil, high levels of UV radiation significantly contribute to disease prevalence, with unprotected sun exposure and a history of sunburns identified as major risk factors [5].

UV exposure occurs not only during recreational activities but also in occupational settings. In Brazil, numerous formal and informal occupations require daily outdoor work, leading to prolonged sun exposure often without adequate protective measures [6]. Early signs of the disease are frequently underestimated, delaying diagnosis and potentially worsening prognosis. Given the relevance of this issue, this study aimed to describe the occurrence of skin neoplasms in a hospital-based sample from a dermatology referral service and to characterize the epidemiological profile and sun exposure patterns among affected individuals.

This study was previously presented as an electronic poster at the 30th Annual Meeting of Dermatologists of the State of São Paulo & Cosmiatry, Laser and Technology Symposium of the Brazilian Society of Dermatology (SBD), held on November 14-15, 2025, at the Frei Caneca Convention Center in São Paulo, Brazil.

## Materials and methods

This exploratory, cross-sectional, descriptive, hospital-based study was conducted at the Dermatology Outpatient Clinic of the Hospital Municipal Universitário de Taubaté (H.MUT), a public referral healthcare center located in Taubaté, São Paulo, Brazil. Data collection took place between December 2024 and June 2025. The study was approved by the Research Ethics Committee of the University of Taubaté (CAAE (*Certificado de Apresentação para Apreciação Ética*): 81852424.9.0000.5501; opinion number: 7.302.324) and conducted in accordance with ethical standards for research involving human subjects.

Sampling and participants

A non-probabilistic convenience sample was used. All adult patients (≥18 years) who attended the Dermatology Outpatient Clinic during the study period and agreed to participate were consecutively included. This approach also allowed the estimation of the occurrence of skin neoplasms in this referral service during the study period. For the final analytical sample, only individuals with a confirmed current or previous diagnosis of skin neoplasms were included, resulting in a case-only descriptive analysis. Individuals diagnosed with other dermatological conditions were not included in the final analytical sample. Diagnoses were verified through medical records and histopathological confirmation. No formal sample size calculation was performed, as the study was exploratory and based on all eligible patients attending the service during the study period.

Inclusion and exclusion criteria

Inclusion criteria comprised adult patients (≥18 years) attending the Dermatology Outpatient Clinic during the study period who consented to participate. For analytical purposes, only patients with a confirmed current or previous diagnosis of skin neoplasms were included. Exclusion criteria included patients younger than 18 years of age and individuals diagnosed with dermatological conditions other than skin neoplasms.

Data collection

Data were collected using a structured questionnaire developed by the authors specifically for this study. The instrument was designed based on variables relevant to occupational sun exposure and skin neoplasm risk, including demographic characteristics (sex, age, skin color, and educational level), occupational history (current and previous occupations), occupational sun exposure (frequency and time of day), photoprotection practices, and personal and family history of skin neoplasms. Continuous variables, such as age, were grouped into predefined intervals, as specified in the questionnaire, for descriptive analysis. Open-ended occupational responses were categorized into indoor and outdoor occupations based on the predominant work environment. Missing or non-reported data were categorized as “not reported” for analysis. The questionnaire consisted of closed-ended and structured questions to allow standardized data collection. Prior to its application, the instrument was reviewed by clinicians with experience in dermatology to ensure content relevance and clarity. However, no formal validation or reliability testing was performed. The complete instrument is provided in the Appendices.

Statistical analysis

Data were entered into and analyzed using Microsoft Excel, version 365 (Microsoft Corporation, Redmond, Washington, United States). Descriptive statistical analyses were performed. Categorical variables are presented as absolute (n) and relative (%) frequencies. Continuous variables were initially recorded and subsequently categorized for descriptive purposes. No inferential statistical analyses were performed due to the exploratory and descriptive nature of the study.

## Results

**Figure 1:**
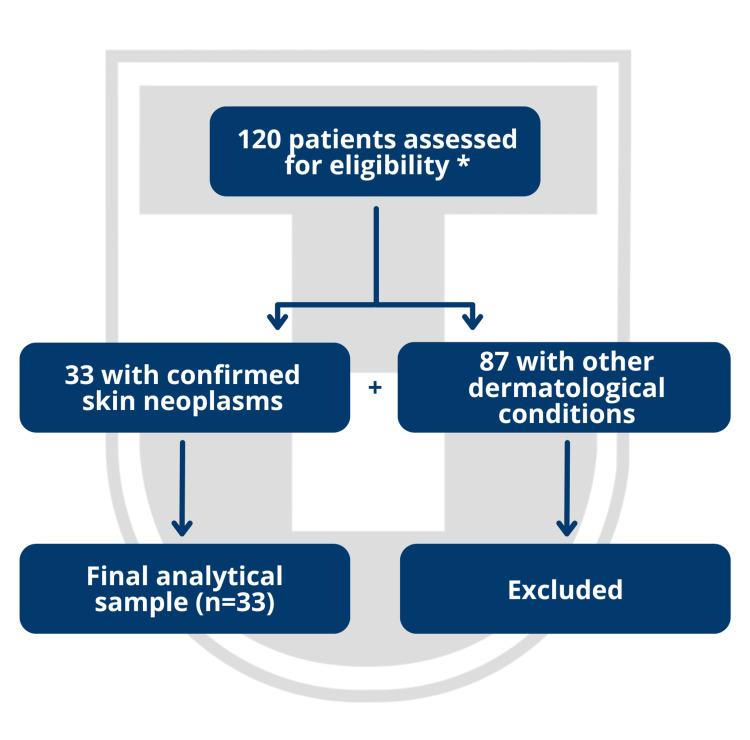
Flow diagram of patient selection and inclusion in the study * All eligible patients were initially included to allow estimation of the occurrence of skin neoplasms in this hospital-based setting during the study period. Only individuals with confirmed diagnoses were retained for the final analysis. Image created using Microsoft PowerPoint (Microsoft Corporation, Redmond, Washington, United States).

A total of 120 individuals were assessed during the study period, of whom 33 (27.5%) had a confirmed diagnosis of skin neoplasms and were included in the final analytical sample, as illustrated by the flow diagram of patient selection and inclusion in Figure 1.

The demographic and clinical characteristics of the sample (n = 33) are presented below. A total of 18 participants (54.5%) were male, and 15 (45.5%) were female. Most participants self-identified as White (n=30, 91.0%) (Table 1).

**Table 1:** Distribution of skin color in the sample (N = 33)

Skin Color	Frequency	Percentage
White	30	91.0
Brown	3	9.0
Yellow	0	0.0
Black	0	0.0

At the time of assessment, 27 participants (82.0%) were older than 55 years, and no participants were younger than 35 years (Table 2). Age at first diagnosis is detailed in Table 3. Although a wide range of ages at diagnosis was observed, most participants were diagnosed after the age of 55 years (n=17, 51.6%), and 23 (69.8%) were older than 45 years at diagnosis.

**Table 2:** Distribution of current age in the sample (N = 33)

Current age (years)	Frequency	Percentage
18–25	0	0.0
26–35	0	0.0
36–45	1	3.0
46–55	5	15.0
>55	27	82.0

**Table 3:** Distribution of age at first skin cancer diagnosis in the sample (N = 33)

Age at first diagnosis (years)	Frequency	Percentage
18–25	1	3.0
26–35	1	3.0
36–45	1	3.0
46–55	6	18.2
>55	17	51.6
Not reported	7	21.2

Educational level is presented in Table 4. A total of 14 participants (42.4%) had not completed primary education, whereas two (6.1%) had completed an undergraduate degree.

**Table 4:** Distribution of educational level in the sample (N = 33)

Educational level	Frequency	Percentage
Illiterate	1	3.0
Incomplete primary education	14	42.4
Completed primary education	9	27.3
Incomplete secondary education	2	6.1
Completed secondary education	3	9.1
Incomplete undergraduate degree	1	3.0
Completed undergraduate degree	2	6.1
Not reported	1	3.0

**Figure 2:**
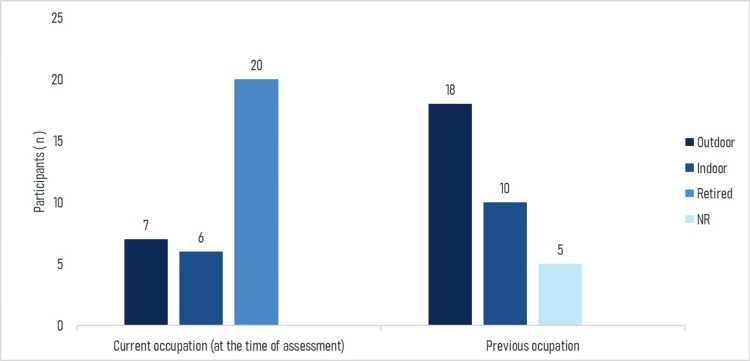
Distribution of current and previous occupational categories in the sample (N = 33) Note: NR indicates not reported. Indoor occupations (e.g., hairdresser, public servant, baker, shopkeeper, waiter, and factory worker) were defined as occupations performed predominantly in enclosed environments, whereas outdoor occupations (e.g., construction worker, farmer, driver, police officer, and gardener) were defined as occupations performed predominantly outdoors. Image created using Microsoft Excel, version 365 (Microsoft Corporation, Redmond, Washington, United States).

A total of 20 participants (60.6%) were retired at the time of assessment. The remaining participants reported various current occupations, including farming, domestic work, construction, and other manual or service-related activities. Previous occupations were predominantly outdoor work (18, 54.5%), such as farming, construction, law enforcement, and driving. A total of five participants (15.2%) did not report their previous occupation. The distribution of occupational categories is presented in Figure 2.

Daily occupational sun exposure was reported by 22 participants (66.6%), with the most commonly reported period being between 10 a.m. and 4 p.m. (16, 48.5%) (Tables 5, 6).

**Table 5:** Distribution of frequency of sun exposure in the sample (N = 33)

Sun exposure frequency	Frequency	Percentage
Every day	22	66.6
3–4 times per week	3	9.0
Once a week	0	0.0
Rarely	1	3.0
No sun exposure	7	21.4

**Table 6:** Distribution of sun exposure according to time of day in the sample (N = 33)

Time of day	Frequency	Percentage
Before 10 a.m.	5	15.1
10 a.m. to 4 p.m.	16	48.5
After 4 p.m.	0	0.0
Before 10 a.m. and after 4 p.m.	4	12.1
All day	5	15.2
No sun exposure	3	9.1

Regarding photoprotection, 12 participants (36.3%) reported daily sunscreen use, one participant (3.0%) initiated sunscreen use only after diagnosis, 14 (42.0%) reported no use, and seven participants (21.2%) reported use only during periods of intense sun exposure. Additional sun-protective measures are presented in Table 7, with hats or caps being the most frequently reported (n=19, 57.6%).

**Table 7:** Distribution of use of sun protective devices in the sample (N = 33)

Use of sun protective devices	Frequency	Percentage
Hat or cap	19	57.6
Long-sleeved shirt	2	6.0
UV-protective shirt	0	0.0
None	9	27.4
Hat/cap, and long-sleeved shirt	3	9.0

Basal cell carcinoma (BCC) was the most frequent histological subtype (n=12, 36.4%), followed by combined BCC and squamous cell carcinoma (SCC) (n=8, 24.3%) and SCC alone (n=7, 21.3%). Combined histological categories were also reported. Melanoma was observed in two cases (6.0%) (Table 8).

**Table 8:** Distribution of histological types in the sample (N = 33)

Histological type	Frequency	Percentage
Not informed	3	9.0
Melanoma	2	6.0
Basal cell carcinoma (BCC)	12	36.4
Squamous cell carcinoma (SCC)	7	21.3
BCC + SCC	8	24.3
BCC + SCC + Melanoma	1	3.0

A positive family history of skin neoplasms was reported by 12 participants (36.0%).

## Discussion

Cancer remains a major global public health concern, with an estimated 20.0% increase in incidence over the last decade, underscoring the need for continuous surveillance to guide prevention and control policies [7]. In Brazil, during 2023-2025, approximately 704,080 new cancer cases were projected, including 8,980 cutaneous melanomas and 220,490 non-melanoma skin neoplasms [8]. Between 2019 and 2023, 399,230 cases of skin neoplasms were registered nationwide, with 46.1% occurring in men and 53.9% in women. This difference may reflect variations in behavioral factors, such as differences in photoprotection practices among men [9].

Among the 33 patients diagnosed with skin neoplasms in this study, 18 (54.5%) were male and 15 (45.5%) were female, slightly differing from the national distribution [8]. Most participants self-identified as White (n=30, 91.0%). This finding is in line with previous data from the same institution (H.MUT), where a study conducted between 2001 and 2005 reported a predominance of White individuals, with a ratio of 4:1 compared to other skin color categories [10].

The relationship between cumulative UV exposure and carcinogenesis is well established, with risk increasing alongside lifetime exposure and decreasing with higher levels of melanin pigmentation [11]. The regional context may further contribute to this risk. Taubaté experiences high solar radiation throughout the year and historically received a significant influx of European immigrants in the late nineteenth and early twentieth centuries for agricultural labor [10]. From a biological perspective, this pattern is consistent with the known relationship between skin phototype and susceptibility to ultraviolet-induced damage [12]. According to the Brazilian Society of Dermatology, the Fitzpatrick scale classifies skin into six phototypes based on response to sun exposure, ranging from very fair skin that always burns to deeply pigmented skin that rarely burns [13]. Individuals with phototypes I-II are more frequently affected, followed by phototype III, whereas phototypes IV-VI present lower incidence rates [14].

It is important to note, however, that skin color in this study was self-reported and does not directly correspond to formal Fitzpatrick phototype classification. In the Brazilian population, characterized by a high degree of ethnic admixture, self-classification into broad categories may be heterogeneous and influenced by sociocultural factors. This may introduce classification bias, as self-reported skin color does not necessarily reflect individual photobiological characteristics relevant to ultraviolet susceptibility. Overall, the distribution observed in this study is consistent with patterns reported in the literature.

Age is a well-established factor in the occurrence of skin neoplasms. Previous studies report a mean age of approximately 64 years among affected individuals [15], consistent with our findings. In the present study, most participants were older than 55 years at both assessment and initial diagnosis. These findings are consistent with the cumulative effects of ultraviolet exposure over time, with risk increasing substantially after the fifth decade of life [16].

Occupational sun exposure represents an important contextual aspect of the population studied. Outdoor and manual occupations, including farming, construction, bricklaying, and driving, were frequently reported among participants, both as current and previous occupations. Previous studies have reported an association between occupational ultraviolet exposure and cutaneous squamous cell carcinoma [17]. Additionally, it has been reported that approximately 65.0% of farmers are exposed to sunlight for more than six hours per day [18]. A similar pattern was observed in our sample, in which 22 of 33 participants (66.6%) reported daily occupational sun exposure, with 16 (48.5%) occurring between 10 a.m. and 4 p.m., when ultraviolet radiation is most intense [19].

Socioeconomic factors may also contribute to vulnerability. In this sample, 14 participants (42.4%) had not completed primary education, whereas only two (6.1%) had completed an undergraduate degree. Similar patterns have been reported in other Brazilian studies [6,20]. Lower educational attainment may be related to differences in access to health information and adherence to preventive measures. 

Preventive behaviors were suboptimal. Only 12 participants (36.3%) reported daily sunscreen use, and one (3.0%) initiated sunscreen use only after diagnosis, suggesting reactive behavior. Meanwhile, 14 (42.0%) reported no use, and seven (21.2%) applied sunscreen only during periods of intense sun exposure. These findings highlight the combined influence of occupational, social, and behavioral factors on skin neoplasm risk and emphasize the need for targeted public health interventions promoting effective photoprotection.

Adherence to non-pharmacological photoprotection measures also appeared limited. In this study, although hats or caps were among the most frequently reported protective measures (n=19, 57.6%), these strategies alone may provide limited protection against ultraviolet radiation, particularly for exposed areas such as the face and neck [21]. More comprehensive approaches, including the use of UV-protective clothing and shade-seeking behaviors, have been described as important strategies to reduce ultraviolet exposure [22]. In occupational settings, Regulatory Standard No. 31 recommends that employers provide protective equipment, including hats, protective clothing, and sunscreen, to workers exposed to sunlight; however, adherence to these measures may vary [23].

BCC was the most frequent histological subtype in our sample (n=12, 36.4%), consistent with its known association with cumulative sun exposure [2]. Combined BCC and SCC and isolated SCC were also observed, whereas melanoma accounted for two cases (6.0%), consistent with expected epidemiological proportions [2]. These findings reinforce the relevance of chronic occupational UV exposure in professions commonly represented in Brazil and reflected in our sample.

Family history was also notable, with 12 of 33 participants (36.0%) reporting a positive familial history of skin neoplasms, consistent with other national studies [6,20]. The interaction of genetic predisposition with environmental and occupational factors underscores the multifactorial nature of skin carcinogenesis and highlights the importance of integrated preventive strategies focusing on education, early diagnosis, and protection of high-risk populations.

Limitations of the study

This study has several limitations. The small sample size (n = 33), the use of a non-probabilistic convenience sample, the hospital-based design from a single referral center, and the cross-sectional design may introduce selection bias, limit the generalizability of the findings, and preclude extrapolation to the general population, as well as causal inferences and longitudinal assessment of disease progression. Additionally, because only individuals with confirmed skin neoplasms who attended the referral service were included in the analytical sample, the study may also be subject to survivor bias, as it does not capture individuals with more aggressive disease who may not have reached or remained in care, nor those without access to specialized services. Furthermore, the study does not allow for the assessment of associations or risk factors, nor comparison with non-affected individuals. Data were obtained through self-report, which may introduce recall and information bias. In addition, occupational sun exposure was not quantitatively measured, which may lead to exposure misclassification. Important potential confounding factors, such as recreational sun exposure, history of sunburns, genetic predisposition, and use of photosensitizing medications, were not controlled for. Furthermore, the questionnaire used in this study was developed by the authors and did not undergo formal validation or reliability testing.

## Conclusions

This study characterizes individuals with skin neoplasms in a hospital-based dermatology referral setting, including their epidemiological profile and patterns of sun exposure. Cases were observed predominantly among older individuals with fair skin, lower educational levels, and a history of outdoor work, with low adherence to photoprotection practices. However, given the study design and sample characteristics, these findings should be interpreted with caution and cannot be generalized to the broader population.

These results may support future research and preventive strategies. Strengthening integrated public health approaches focused on education, prevention, and regulatory compliance is essential, particularly through primary health care interventions, enforcement of occupational safety standards, and the generation of local epidemiological evidence to support more equitable and effective prevention.

## Appendices

Study questionnaire

**Table 9:** Structured questionnaire used for data collection

Parameter	Question	Response Options
Sex	What is your sex?	☐ Female ☐ Male ☐ Prefer not to disclose
Age group	What is your age?	☐ 18–25 years ☐ 26–35 years ☐ 36–45 years ☐ 46–55 years ☐ Over 55 years
Skin color	Self-identified skin color	☐ White ☐ Brown ☐ Yellow ☐ Black
Educational level	What is your highest educational level?	☐ Illiterate ☐ Incomplete primary education ☐ Completed primary education ☐ Incomplete secondary education ☐ Completed secondary education ☐ Incomplete undergraduate degree ☐ Completed undergraduate degree ☐ Not reported
Current occupation	What is your current occupation?	Open-ended response: __________________________
Previous occupation	What was your previous occupation?	Open-ended response: __________________________
Occupational sun exposure frequency	When working outdoors, were you exposed to sunlight?	☐ Yes, every day ☐ Yes, 3 to 4 times per week ☐ Yes, once a week ☐ Yes, rarely ☐ No
Time of sun exposure	When working outdoors, what was the time of sun exposure? (Check more than one option if necessary)	☐ Before 10 a.m. ☐ Between 10 a.m. and 4 p.m. ☐ After 4 p.m. ☐ No sun exposure
Sunscreen use	Do you use sunscreen? How often?	☐ Yes, every day ☐ Yes, only when sunbathing ☐ No, I do not use it
Other sun protection measures	Do you use any other sun protection measures? (Check more than one option if necessary)	☐ Hat or cap ☐ Long-sleeved shirt ☐ UV-protective shirt ☐ Other: ____________________ ☐ None
Skin cancer diagnosis	Have you ever been diagnosed with skin cancer? If yes, which type? (Check more than one option if necessary)	☐ No ☐ Yes, but I do not know the type ☐ Yes, melanoma ☐ Yes, basal cell carcinoma (BCC) ☐ Yes, squamous cell carcinoma (SCC) ☐ Yes, other: ____________________
Age at first diagnosis	If yes, what was your age at the time of the first skin cancer diagnosis?	Open-ended response: __________________________
Family history	Do you have a family history of skin cancer?	☐ Yes, in father, mother, or sibling ☐ Yes, in uncle, aunt, grandfather, or grandmother ☐ Yes, in more distant relatives ☐ No ☐ I do not know
